# Hospital-Acquired SARS-Cov-2 Infections in Patients: Inevitable Conditions or Medical Malpractice?

**DOI:** 10.3390/ijerph18020489

**Published:** 2021-01-09

**Authors:** Rosario Barranco, Luca Vallega Bernucci Du Tremoul, Francesco Ventura

**Affiliations:** Department of Legal and Forensic Medicine, University of Genova, 16132 Genova, Italy; luca.vallega@hotmail.it (L.V.B.D.T.); francesco.ventura@unige.it (F.V.)

**Keywords:** COVID-19, hospital-acquired infection, legal medicine

## Abstract

Despite numerous measures to contain the infection and limit its spread, cases of SARS-CoV-2 infections acquired in hospitals have been reported consistently. In this paper, we will address issues of hospital-acquired COVID-19 in hospitalized patients as well as medico-legal implications. After having conducted a literature search, we will report on papers on hospital-acquired SARS-CoV-2 infections. Ten scientific papers were selected and considered suitable for further analysis. According to several reports, the SARS-CoV-2 hospital-acquired infection rate is 12–15%. Hospital-acquired COVID-19 represents a serious public health issue, which is a problem that could create reluctance of patients to seek hospital treatment for fear of becoming infected. Healthcare personnel should do all that is necessary to address the problem and prevent further spreading, such as rigorous compliance with all procedures for containing the spread. From a medical-legal point of view, multiple aspects must be considered in order to understand whether the infection is a result of “malpractice” or an inevitable condition.

## 1. Introduction

The first cases of SARS-CoV-2 were reported in Wuhan, the capital of the Hubei Province in China, in December 2019 and it has rapidly spread all over the globe. It has become an international public health issue [1]. The World Health Organization deemed the new Coronavirus 2 respiratory infection (COVID-19—Coronavirus disease 2019) a pandemic in March 2020 [2].

The SARS-CoV-2 disease has placed a strain on governments of all nations as well as health care personnel in their attempt to reduce infections, find an effective cure for patients, and prepare a vaccine [3].

Typically, if a person comes into close contact with infected people, respiratory infections can be transmitted through exposure to droplets [4]. The incubation period is generally between three and seven days with a range of 2–14 days [5,6]. The clinical course of COVID-19 varies considerably, ranging from completely asymptomatic cases to fatal pneumonia [7]. In the early stages, general symptoms can be fever, fatigue, cough, myalgia, and dyspnoea while minor symptoms include dizziness, headaches, nausea, diarrhea, and vomiting [8,9].

Dyspnoea and hypoxia are major symptoms that may occur within a few days (approximately one week) after the onset. In seriously ill patients, acute respiratory distress syndrome, metabolic acidosis, coagulation dysfunction, and septic shock can occur quickly and cause death [2,10].

Characteristics of SARS-CoV-2 are the ease of contagion as well as evidence that the virus can remain active on inanimate surfaces for up to three days [4,6].

As well as person-to-person transmission and social activity, it is apparent that so-called super-diffusion events in the hospital setting are responsible for continued outbreaks and clusters. Prevention strategies and practical measures, such as isolation of the infected, containment of severe epidemic areas, and the tracing and quarantine of close contacts are necessary to slow the spread of the virus and reduce infections. Early detection and diagnosis of patients with COVID-19 can lead to a better prognosis and reduction of the infection spread [11].

Despite numerous measures to contain the infection and prevent contagions, cases of Coronavirus 2 infections acquired in hospitals have been reported [4,12]. The latter has brought negative repercussions in public health, risk management, the control of hospital-acquired infections (HAIs), and in the medico-legal field.

In this paper, we discuss hospital-acquired SARS-CoV-2 infections as well as their medico-legal implications. After having conducted a literature search, we will report on papers, case series, and reviews on hospital-acquired SARS-CoV-2 infections.

## 2. Literature Research 

All scientific papers regarding hospital-acquired COVID-19 infections were reviewed. Specifically, search engines MEDLINE, EMBASE, PUMED, and Scopus were used for the search of keywords (search formula): “COVID-19” or “coronavirus disease 2019” or “SARS-CoV-2” or “severe acute respiratory syndrome-coronavirus 2” or “Novel CoV” or “2019-nCoV” “2019 novel coronavirus” and “hospital infection” or “hospital-acquired infection”. Additional “preprint” reports were searched as well.

We have included all articles that involved cases of COVID-19 acquired in the hospitals by patients. Articles that dealt with infections arising in health care workers were not included (the articles specifically referred to occupational and work-related diseases). Only articles in the English language were included in the analysis.

The authors first analyzed the abstracts and then thoroughly reviewed the articles.

## 3. Literature Data

Five searches were conducted. Articles relating to hospital-acquired SARS-CoV-2 infections were selected. After an analysis of the search engine results, the titles and abstracts of articles were examined. Articles that did not relate to the topic were not included in the study. After a preliminary analysis, a total of 23 articles were selected. Thirteen articles that reported COVID-19 among healthcare workers were not considered. The search yielded 10 scientific papers considered suitable for analysis. The following is a summarization of the selected papers (Table 1).

In the first article (preprints with The Lancet), Marago et al. [13] analyzed the prevalence of hospital-acquired COVID-19 and evaluated whether patient characteristics had influenced the onset of the infections. A retrospective case analysis of the General District Hospital in the North West of England was carried out. A total of 239 patients tested positive for COVID-19 with a percentage of hospital-acquired cases reaching 16.2%. According to the authors, patients with hospital-acquired infections endured longer hospital stays.

In a study published in JAMA, Wang et al. [14] described a case series of 138 patients with 2019 novel coronavirus pneumonia. Hospital-associated transmission was suspected in 12.3% of hospitalized patients (17 cases), initially admitted for other health issues. Of the hospitalized patients, five were from internal medicine, seven from the surgical ward, and five from the oncology ward. Overall, 26% of the patients received intensive care unit care. Patient-to-patient transmission (as a result of inadequate isolation) was considered the cause of infection in several cases. Data and differences regarding the clinical evolution between hospital-acquired and community infections were not indicated.

Tani et al. [15] carried out a study of Japanese articles from 15 February to 6 April 2020. According to this study, a total of 246 HAIs had occurred in 17 medical institutions, in 10 prefectures. These cases accounted for 6.9% of Japan’s total. The number of infected cases in each hospital varied greatly and ranged from 1 to 128. According to the authors, the causes of HAIs were mainly due to insufficient isolation of the infected and inadequate personal protection.

Zhou et al. [16] carried out a review and meta-analysis of cases in China-based databases of HAIs in patients with COVID-19, SARS, and MERS. Of the 40 studies concerning SARS-CoV-2, only four were included [14,17,18,19]. According to the study, the overall proportion of Coronavirus 2 infections contracted in hospitals (in all cases, including health care workers) reached 44%. Most of the confirmed patients were medical personnel and other HAIs, which accounted for 33.0% and 2.0% of COVID-19 cases, 19.0% and 36.0% of MERS cases, and 37.0% and 24.0% of SARS cases, respectively. Nurses and doctors were the most affected among the medical personnel. Only 2% regarded hospitalized patients (inpatients) and visitors.

Rickman et al. [20] performed a retrospective study on 66 hospital-acquired cases at a University Hospital in London. According to the study, 15% (66/435) of COVID-19 cases (recorded between 2nd March and 12th April 2020) were either definitely or probably hospital-acquired. Specifically, of the 435 COVID-19 positive patients, 47 (11%) were classified as “definite hospital-acquired” and 19 (4%) were defined as “probable hospital-acquired”. The mortality rate was 36%. Regarding transmission, the authors reported that 36 cases (55%) had been identified as having been located in the same bay as patients with COVID-19 during a period compatible with the incubation period. Nine of the cases (14%) had no contact in the same bay but within the same ward, while 21 cases (32%) did not indicate evident sources of infections (with other positive patients).

Carter et al. [4] conducted an observational study of COVID-19 cases acquired in United Kingdom hospitals. Of the 1564 patients admitted, 12.5% of the COVID-19 infections were hospital-acquired. The median survival time in patients with hospital-acquired SARS-COV-2 infection was 14 days compared with 10 days in community-acquired COVID-19 infection. The mortality rate was 27.2% with a lower rate in hospital-acquired infections (27%). Hospital-acquired COVID-19 infection patients required extended hospital recovery time during convalescence.

Cheng et al. [21] analyzed cases of COVID-19 during the pre-pandemic phase in Hong Kong. The data revealed 130 confirmed cases of SARS-CoV-2 infections and no cases of hospital-acquired transmission. According to the authors, “On day 85, the number of confirmed cases of SARS-CoV-2 was over 300 in Hong Kong while zero transmission of SARS-CoV-2 among HCWs and hospitalized patients could be achieved”.

Iacobucci [22] revealed problems linked to the hospital transmission of the virus, reporting the opinions of several doctors as well as unofficial data from the news media. According to his paper, health care professionals were concerned about the number of patients who had been infected in NHS hospitals in England, stressing the importance of very stringent measures for containing the infection spread in hospitals. In addition, there were several confirmed cases of patients being infected by other patients while hospitalized and subsequently died. The paper does not report official data; however, according to *The Guardian* newspaper, the hospital infection rate would be between 10 and 20% although NHS sources report these data as being overestimated and that the correct percentage is 5–7% [23]. The paper emphasizes the importance of timely testing of all patients and strict isolation in order to reduce the infection spread.

Rhee et al. [24] analyzed hospital-acquired COVID-19 at a United States Academic Medical Center. According to the cohort study, “9149 inpatients were admitted to a large US academic medical center for a period of 12 weeks. Six hundred and ninety-seven were diagnosed with COVID-19. Only two hospital-acquired cases were detected: one patient was likely infected by a presymptomatic spouse before visitor restrictions were implemented, and one patient developed symptoms 4 days after a 16-day hospitalization but with no known hospital exposure”.

Jewkes et al. [25] reported on the spread of COVID-19 in a Birmingham Neurology ward during the first phase of the pandemic. According to the authors, 21 of the 133 admissions (16%) were COIVD-19 positive. Eight of the admissions (approximately 6%) were healthcare-related infections. Ten SARS-CoV-2 infected patients had died.

## 4. Discussion

HAIs emerged in healthcare settings where patients were hospitalized for reasons unrelated to infections or were not previously infected prior to the admission [26]. Although the number of HAIs has increased dramatically over the past 30 years, a proportion of HAIs can be considered preventable with an accurate public health surveillance system, infection control, best practices, and prevention [27]. The prevalence of HAIs in high-income nations is about 7.5%. However, prevalence rates of 5.7–7.1% in Europe and 4.5% in the United States have been reported. In contrast, rates in low-income countries vary from 5.7 to 19.2% [28,29,30,31]. The rate varies considerably from country to country according to infection control and prevention measures [28].

HAIs have been revealed to be a major hospital problem due to morbidity, mortality, an increase in hospitalization duration, and costs [32].

Older age constitutes a predisposing factor to HAIs. Individual susceptibility to infection increases greatly for those with serious concomitant pathologies. HAIs occurred in four main localizations and represent the majority of all infections: respiratory, urinary tract (UTI), surgical wounds, and systemic infections [33,34]. At present, antibiotic resistance represents a major concern with regard to HAIs [35]. In addition to bacteria and fungi, viruses are also a cause of HAIs. Generally, hospital virus infections can be transmitted through the respiratory route, hand–mouth contact, and fecal–oral contact route [33,36,37,38].

According to estimates, a part of HAIs can be preventable if measures of prevention such as specific healthcare practices, adequate professional behavior, and correct organizational structure are adopted as well as strict compliance with guidelines of prevention [34,39]. However, a part of HAIs would still remain unpreventable due to external factors (relating to the patient or medical treatment itself). Infectious complications related to medical care will have an impact on economic costs; therefore, HAIs represent an important factor of increased healthcare costs [40] due to multiple aspects:

- Delay in hospital discharges with an increase in hospitalization costs;
- Increase in treatment costs;
- Increase in the number of diagnostic and laboratory investigations;
- Medico-Legal disputes.

According to a United States analysis, hospital costs related to HAIs are very high [41]. Another study revealed that hospital costs for each HAI had increased by about 12,000 US dollars [42].

There are no measures that can completely eliminate the risk of hospital infection, however, there are several that can be adopted to decrease its incidence and severity.

Prevention of healthcare-related infections requires increased personal hygiene and environmental proceedings (healthcare personnel frequently washing their hands as well as the use of personal protective equipment, such as masks, overshoes, gowns, gloves, to contain the transmission of infectious agents). Furthermore, correct patient management is required with the immediate adoption of isolation procedures [43].

In the present brief article, the occurrence of nosocomial COVID-19 as described in the literature has been analyzed.

Based on the results of the study, hospital-acquired SARS-CoV-2 infections are an important issue. According to official data of several published studies [4,14,20], a rate of 12–15% of nosocomial COVID-19 in patients has been reported.

According to the meta-analysis by Zhou et al. [15], the rate of HAIs (of inpatients) is 2% but the overall proportion of Coronavirus 2 infections contracted in hospitals (all cases, including healthcare personnel) is 44%. Notwithstanding, in other studies such as the one by Rhee et al. [24], the incidence of hospital-acquired COVID-19 is low and negligible.

An explanation of these differences is not simple as well as a number of factors that may be associated: socio-demographic context, the lack of individual protective equipment and healthcare personnel, and the overcrowding of hospitals. The increased number of parameters to be taken into consideration and a limited understanding of the virus has proven difficult in obtaining a complete evaluation.

The high number of HAIs refers to the first wave of the pandemic when hospitals were still unaware of how to manage the new global pandemic and individual prevention equipment was still insufficient. Compared to other reported rates of HAIs during previous global pandemics, it appears that the pandemic rates of COVID-19 are much lower [4,20].

The countries with the highest number of SARS-CoV-2 infections were the first to be “struck” by the pandemic (such as China, Italy, and the UK). It is possible that the hospitals in these countries found themselves “unprepared” to manage the emergency. Instead, countries that were stricken afterward had ample time and knowledge to prepare the resources needed to manage the emergency. This may have allowed for the timely diagnosis of COVID-19 cases, the proper isolation in dedicated “COVID-19” wards, and the use of efficient measures of individual protection [4].

The main reasons behind the nosocomial spread were the incorrect isolation, the use of shared healthcare equipment, and the constant movements of infected personnel, (a particularly serious and widespread problem especially during the first wave of the pandemic) [16,20,44].

Regarding mortality, the rate was about 36%, according to Rickman [20], while according to Carter et al. [4], there were no significant variations in rate between hospital-acquired and community-acquired infections.

Since patients with hospital-acquired COVID-19 are generally elderly, frail, and suffer from other health issues, the increase in mortality would theoretically be expected. Nevertheless, hospitalized patients are usually constantly evaluated and therefore the infection was probably identified and diagnosed early. Patients with community-acquired COVID-19 may have tolerated the symptoms at home for some time before hospitalization, and a diagnosis may have been affirmed at a point when the disease became severe (unlike patients already hospitalized) [4].

During the first phase of the pandemic, healthcare professionals unknowingly played a role in the spread of the infection. During the first months, they were confronted with the difficult situation of managing a rare and dangerous reality. The shortage of individual protective devices, the incorrect implementation of distancing measures, and work overload have favored the spread of the infection among healthcare personnel and patients. In fact, the progress that has been achieved in recent months has reduced risks. Improvements include optimized triage systems, greater knowledge of transmission and the role of asymptomatic and presymptomatic infections, better access to effective personal protective equipment, improved testing capabilities, implementation of new contagion prevention measures such as the continued use of masks in hospitals [45].

From a medico-legal point of view, COVID-19 has brought significant repercussions and implications [46,47]. Hospital-acquired COVID-19 from nosocomial transmission could probably be a topic of medico-legal dispute seeing that the hospitals and the personnel could be held responsible. It is important to know if healthcare professionals had implemented all the necessary measures to prevent the risk of contagion and if the infection could have been “inevitable” (not due to errors by healthcare personnel) or “avoidable” (related to healthcare responsibility). Specifically, HAIs require an investigation of how an infection occurred and whether it could have been avoided through measures to prevent infectious risk [48]. Compliance with all protective procedures is fundamental when it comes to professional liability claims. On the contrary, failure to comply with these measures is often viewed as negligence and medical fault [48]. In order to determine the absence of professional healthcare responsibility, it is necessary to demonstrate the complete application of all the measures of prevention indicated in the scientific literature as well as current regulatory provisions [49].

During the first stage of the pandemic, nosocomial transmission could have been considered “inevitable” due to the reality that healthcare workers were facing an emergency never experienced before and hospitals often lacked space, equipment, and supplies to handle the emergency. What could have been done to avoid hospital-acquired infections given the overcrowded hospitals (due to the quickly elevated number of patients), insufficient protective equipment and tests, and overworked healthcare personnel? Probably little or nothing. In situations of absolute emergency, even the utmost diligence and care are not sufficient due to the insurmountable difficulties of healthcare management.

During an emergency, it is not easy to determine and track down malpractice. Full compliance with guidelines has proven very difficult due to a lack of resources.

With time, the scientific community began to understand how to effectively confront the pandemic. Prevention strategies were validated and forms of protection and early diagnosis (individual protective equipment, tests, tracking, and correct isolation) had become sufficient. Cases of hospital-acquired COVID-19 should be considered unexpected events that require a thorough analysis of medical records in order to determine what the miscalculations were. We must verify at what moment of the hospitalization did the infection occur, if a correct screening was performed and if there was a “failure” of the measures to prevent the risk of contagion.

It is very difficult to provide general indications. Multiple aspects must be considered to understand if the infection is due to malpractice or inevitable conditions. We need to understand if hospitals and personnel were overloaded and if facilities were properly set up for isolating patients. We must understand if healthcare personnel were given additional resources necessary for the prevention of contagion. Work complications (work overload, shortage of personnel, shortage of isolation space, etc.) will not allow contagion prevention measures to be correctly and promptly followed. Therefore, a maximum commitment from health professionals cannot be considered sufficient. However, fault cannot be placed solely on healthcare workers. Given the complicated situation, a hospital-acquired infection should not be considered grounds for "malpractice".

However, if containment measures are not respected due to an "error", then a hospital infection could be considered “malpractice”. Failure to follow infection control standard precautions would be sufficient proof of professional liability.

Hospital-acquired COVID-19 cases require a complete analysis of medical record documentation. Medico-legal evaluation is very complex and must take into account individual, organizational, and healthcare facility factors.

## 5. Conclusions

Hospital-acquired COVID-19 represents a serious public health problem and the first articles indicate there was an alarming rate of HAIs. It is a problem that could cause reluctance of patients to seek hospital care for fear of becoming infected. Scientific studies have proven that in-hospital transmission of COVID-19 is not negligible. According to several reports, the hospital-acquired SARS-CoV-2 infection rate is 12–15%. Patients admitted to hospitals must give undivided attention to individual protection measures. Healthcare personnel must do all that is possible to address the problem and prevent further spreading, through rigorous compliance with procedures for containing the infection.

The reporting of these events and in-depth hospital investigations are necessary to fully understand the origin of the infection, implement corrective measures, and prevent an increase in cases.

## Figures and Tables

**Table 1:** Parameters of selected studies: country, observation period, and percentage of hospital-acquired SARS-CoV-2 infections.

Authors	Country	Date	Percentage
Marago et al. [13]	North West of England	May 2020	16.2%
Wang et al. [14]	Wuhan, China	January/February 2020	12.3%
Tani et al. [15]	Japan	February/April 2020	6.9%
Rickman et al. [20]	London	March/April 2020	15%
Carter et al. [4]	United Kingdom and Italy	Up to April 2020	12.5%
Cheng et al. [21]	Hong Kong	First 72 days after announcement of pneumonia cases in Wuhan	0
Iacobucci et al. [22]	England	-	5–7%
Rhee et al. [24]	Boston, Massachusetts	March/May 2020	2 cases out of 697 COVID-19 patients
Jewkes et al. [25]	Birmingham, England	March/May 2020	8%

## Data Availability

Not applicable.
